# Multiband Spectrum Sensing and Power Allocation for aCognitive Radio-Enabled Smart Grid

**DOI:** 10.3390/s21248384

**Published:** 2021-12-15

**Authors:** Jun Wang, Weibin Jiang, Hongjun Wang, Yanwei Huang, Riqing Chen, Ruiquan Lin

**Affiliations:** 1 College of Electrical Engineering and Automation, Fuzhou University, Fuzhou 350116, China; wangjunfzu@fzu.edu.cn (J.W.); 200120078@fzu.edu.cn (W.J.); sjtu_huanghao@fzu.edu.cn (Y.H.); rqlin@fzu.edu.cn (R.L.); 2 College of Electronic Engineering, National University of Defense Technology, Hefei 230037, China; 3 College of Computer and Information Sciences, Fujian Agriculture and Forestry University, Fuzhou 350002, China; riqing.chen@fafu.edu.cn

**Keywords:** cognitive radio, smart grid, spectrum sensing, power allocation

## Abstract

As part of an Internet of Things (IoT) framework, the Smart Grid (SG) relies on advanced communication technologies for efficient energy management and utilization. Cognitive Radio (CR), which allows Secondary Users (SUs) to opportunistically access and use the spectrum bands owned by Primary Users (PUs), is regarded as the key technology of the next-generation wireless communication. With the assistance of CR technology, the quality of communication in the SG could be improved. In this paper, based on a hybrid CR-enabled SG communication network, a new system architecture for multiband-CR-enabled SG communication is proposed. Then, some optimization mathematical models are also proposed to jointly find the optimal sensing time and the optimal power allocation strategy. By using convex optimization techniques, several optimal methods are proposed to maximize the data rate of multiband-CR-enabled SG while considering the minimum detection probabilities to the active PUs. Finally, simulations are presented to show the validity of the proposed methods.

## 1. Introduction

The Internet of Things (IoT) has the power to reshape the world as we know it. The Smart Grid (SG), as part of an IoT framework, adopts advanced Information and Communication Technologies (ICTs) [1,2] to constantly optimize electrical power generation, delivery, consumption, and storage of electricity. In order to achieve this goal, SG applications need to transmit a huge amount of data, such as meter readings, sensor data, surveillance data, multimedia data, automation data, and services [3], so effective communication is very important for SG applications.

Wireless communication technologies are strongly recommended for SG applications [4,5], because of their flexibility, wide coverages, wall-penetration capabilities, and ease of installation. However, as mentioned before, considering a huge amount of data is needed to be shared within the SG information network and the spectrum limitations of existing wireless communication technologies, advanced communication technologies are extremely needed for SG applications.

Cognitive Radio (CR) is a wireless communication paradigm in which the unlicensed users or Secondary Users (SUs) who have no spectrum licenses can opportunistically access and use the unused spectrum of licensed users or Primary Users (PUs) who have spectrum licenses without causing interference to the PUs. In a typical CR system, SUs first perform spectrum sensing to find the unused bands and then adjust their transmission parameters such as coding schemes, modulation schemes, and transmitting power to access the unused bands. There are three types of spectrum-sharing techniques in CR systems: interweave, underlay, and overlay [6]. In interweave CR systems, SUs can only use the unused bands of PUs, and in underlay CR systems, SUs can use the bands below certain power limits without causing interference to PUs. In overlay CR systems, SUs actively help in PUs’ data transmission. However, PUs should share knowledge of their signal codebooks and messages with SUs in order to cooperate with each other. In this paper, we assumed there is no cooperative communication between PUs and SUs. Hence, only interweave and underlay CR systems were taken into account.

There are already several papers about how to incorporate CR technology into SG to improve the data rate. References. [7,8] proposed a novel idea of a hybrid CR-enabled SG communication network considering the unpredictable activities of PUs. Because of the unknown statuses of PUs, the unlicensed bands may be very difficult to access for SUs. Therefore, the communication links may become very unstable when PUs have high probabilities of appearance. Therefore, the traditional CR communication network may not be very suitable for SG communication because many SG applications such as Distribution Automation (DA) and real-time Demand Response Management (DRM) extremely need reliable communication links. The novel idea of the hybrid communication network proposed in [7,8] consists of not only the unlicensed bands, which are shared with PUs and SUs, but also some licensed bands, which are bought from the telecommunication operators. Unlike the unlicensed bands, the licensed bands can be accessed all the time by SG users with no need for spectrum sensing. In this way, the communication links in the hybrid network are more reliable than the communication links in the traditional CR network. Based on this novel hybrid network, Reference [9] proposed a sensing-performance trade-off metric to optimize the sensing time while maximizing the DRM performance in a CR-enabled SG communication network, which contains a single licensed band and a single unlicensed band. Reference [10] further proposed several joint spatial and temporal spectrum sharing methods to optimize the DRM performance for a single-band-based CR-enabled SG network. However, in this literature, nothing was mentioned about how to find the optimal sensing time and power allocation scheme simultaneously in such a hybrid CR-enabled SG network, which is a fundamental task in CR.

Moreover, multiband CR technology is usually superior to single-band CR technology because multiple bands can offer more access opportunities for SUs. Thus, multiband CR can significantly improve the data rate of a communication system. As a result, multiband spectrum sensing is a hot research topic in traditional CR networks [11]. Reference [12] first proposed an optimal multiband spectrum sensing for CR, but without considering the optimization of sensing time. Reference [13] further proposed an optimal multiband spectrum sensing and resource allocation method. Based on [13], Reference [14] proposed a joint multiband cooperative spectrum sensing and resource allocation framework for Internet of Things (IoT) in cognitive 5G networks. Reference [15] proposed a real-time implementation method of multiband spectrum sensing based on SDR technology. However, References [12,13,14,15] were all based on the traditional CR network and were not based on the idea of the hybrid CR-enabled SG communication network proposed in [7,8]. Therefore, the methods proposed in [12,13,14,15] may not be very reliable for SG applications because their communication links can be interrupted by the PUs according to the above.

In this paper, based on the new idea of the hybrid CR-enabled SG communication network, which comprises both unlicensed bands shared by PUs and licensed bands bought from the telecommunication operators, we first propose a system architecture for multiband-CR-enabled SG communication and some optimization mathematical models to jointly find the optimal sensing time and the optimal power allocation strategy. Then, by using convex optimization techniques, several optimal methods are proposed for both interweave and underlay multiband-CR-enabled SG to maximize the total data rate. Finally, we demonstrate the effects of the proposed methods by presenting some simulation results to prove the soundness of our proposed algorithms.

The remainder of this paper is organized as follows: Section 2 introduces the backgrounds and the system model of the proposed methods. Section 3 introduces the joint spectrum sensing and power allocation method for interweave-CR-enabled SG. Section 4 gives the joint spectrum sensing and power allocation method for underlay-CR-enabled SG. Section 5 presents the simulation results, and conclusions are made in Section 6.

## 2. Background

### 2.1. Energy Detection

Energy detection is the most often considered spectrum sensing method in the CR literature because of its simplicity and adequate performance. The received signal y(n) of energy detection is [16]:

(1) $$y\left(n\right)=\{\begin{array}{cc} w(n) & H_{0}\\ hx(n)+w(n) & H_{1} \end{array}$$

where x(n) is the PU’s signal, w(n) is the additive white Gaussian noise with zero mean and variance $\sigma_{w}^{2}$, *h* is the channel gain and assumed to vary with time, but remains invariant during one frame, and *n* denotes the *n*-th sample of *N* total samples. The test statistic of energy detection is:

(2) $$\eta =\frac{\sum_{n=1}^{N}y^{2}\left(n\right)}{N}$$

In CR, the main concern is that the presences of PUs should be detected properly and the transmission of PUs should not be interfered by SUs. Thus, the detection probability should be considered first. The false alarm probability means the loss of access opportunities and will cause no harm to PUs so that it can be considered second. Based on this premise, in this paper, we chose the detection probability as the target detection probability. Therefore, given the target detection probability $P_{d0}$, the threshold λ and the false alarm probability $P_{fa}$ can be obtained as:

(3) $$\lambda =2\sigma_{w}^{2}erfc^{-1}\left(2P_{d0}\right)\sqrt{\frac{1+2\gamma }{N}}+\sigma_{w}^{2}+\sigma_{w}^{2}\gamma$$

(4) $$P_{fa}=\frac{1}{2}erfc\left(erfc^{-1}\left(2P_{d0}\right)\sqrt{1+2\gamma }+\frac{\gamma \sqrt{N}}{2}\right)$$

where erfc(.) or $erfc^{-1}\left(.\right)$ is the complementary error function or inverse complementary error function, respectively, and $\gamma \overset{\Delta }{\;=}\sum_{n=1}^{N}\frac{h^{2}x^{2}\left(n\right)}{N\sigma_{w}^{2}}$ is the Signal-to-Noise Ratio (SNR).

### 2.2. System Model

The CR-enabled SG network architecture can be divided into three layers, that is the Home Area Network (HAN), Neighborhood Area Network (NAN), and Wide Area Network (WAN) [17]. The HAN consists of various kinds of smart devices equipped with sensors and a smart meter used as a Home Gateway (HGW) in home energy management systems. The smart meter communicates with the smart devices to monitor, control, and manage the energy efficiently. The NAN covers the distribution and transmission domains and communicates with the service providers in the WAN and the HGWs in the HAN. In order to support SG applications such as power outage management, power quality monitoring, and distribution automation, the NAN may cover several square kilometers and needs at least 10 Mb/s to keep a good connection with a few hundred to a few thousand HGWs [7]. The WAN covers the transmission and power generation domains and communicates with multiple NANs and the control center. A very high volume of data, including the data of power generation plants, control centers, substations, transmission and distribution grids, and distributed energy resource stations, may be communicated via WAN links. Considering a vast number of heterogeneous smart devices and the different types of network traffic, it is a challenging task to guarantee the reliable communication links for the HAN, NAN, and WAN, without more advanced communication technologies.

**Figure 1:**
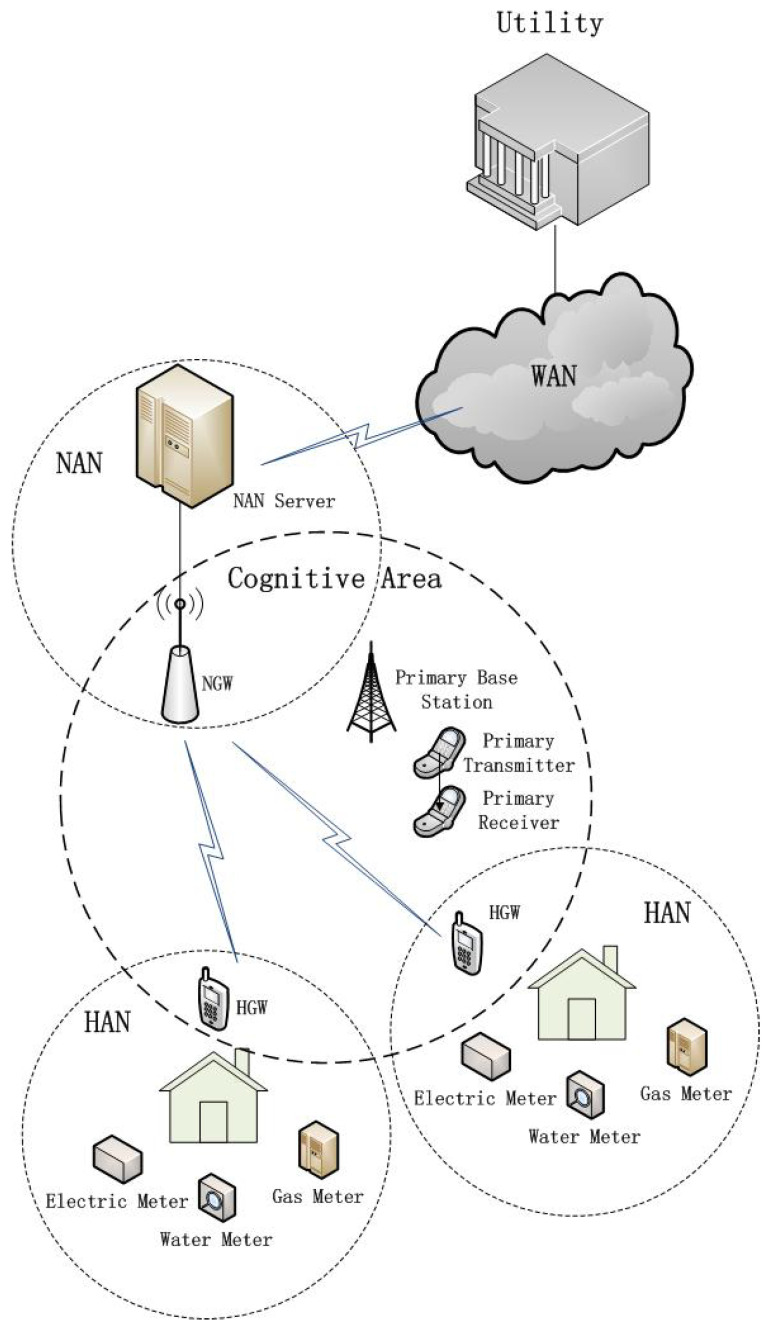
Architecture of the CR-enabled SG communication network.

Because of the dynamic spectrum-sharing mechanism of CR, the CR-based SG emerges as a promising technology to meet the complex communication needs of the HAN, NAN, and WAN. For example, as illustrated in Figure 1, the HGWs collect the data from the electric meters, the water meters, and the gas meters and then transmit the data to the NAN Gateway (NGW). Then, CR technology can be adopted by the HGWs and NGWs if the PUs allow spectrum sharing in the cognitive area. In Figure 1, SG users (HGWs and NGWs) are SUs and try to access the unused spectrum bands of PUs. However, due to the unpredictable activities of PUs, the communication links absolutely dependent on CR are unreliable. Thus, researchers suggest that the SG users buy some additional licensed bands from the telecommunication operators, which may be narrower than the unlicensed bands of PUs because of the expensive spectrum purchase cost, but can be used by the SG users all the time [7]. These licensed bands owned by the SG users are called original channels in this paper. The SG users can also access the unlicensed bands owned by the PUs via CR technology. These bands that are unlicensed to the SG users are called cognitive channels in this paper. These unlicensed or cognitive channels may have wide bandwidths, but can only be accessed and used before proper spectrum sensing, while the licensed or original channels may have narrow bandwidths, but can be accessed and used all the time.

**Figure 2:**
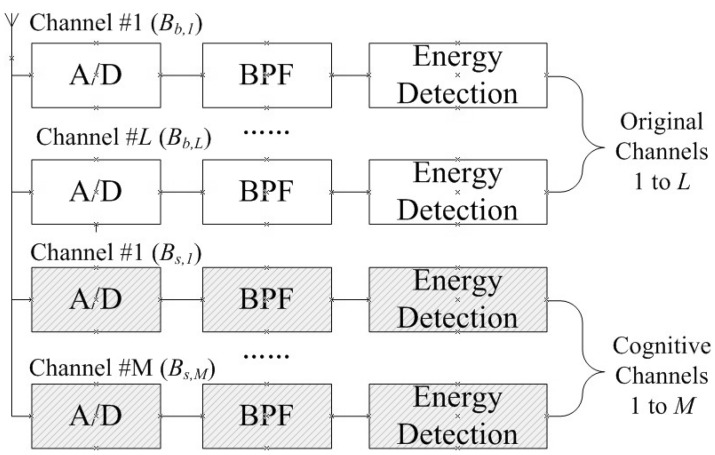
Block diagram of the multiband SG user’s receiver.

According to the above, assume the CR-enabled SG communication network consists of *L* original channels with bandwidths $B_{b,1},\ldots ,B_{b,L}$, which are bought from telecommunication operators, and *M* cognitive channels with bandwidths $B_{s,1},\ldots ,B_{s,M}$, which are shared by PUs. The SG users can access the original channels all the time, but can only access the cognitive channels when PUs are absent or the interference limits are not exceeded. Assume that the SG users can transmit data over the *L* original channels and *M* cognitive channels simultaneously by some advanced communication technologies such as Carrier Aggregation (CA) and Orthogonal Frequency Division Multiplexing (OFDM) during the transmission period. The block diagram of the proposed single-antenna multiband system is shown in Figure 2, where A/D means an Analog-to-Digital converter and BPF means a Band-Pass Filter. Moreover, all channels share one antenna, which means they should perform transmission and reception at the same time.

**Figure 3:**
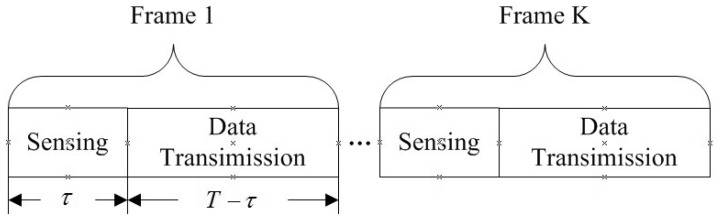
Frame structure.

The time frame with length *T* is divided into two slots: spectrum-sensing slot with length τ and transmission slot with length T−τ. Assume the sampling interval is $T_{s}$, then the number of sensing samples *N* is $T/T_{s}$. The frame structure is shown in Figure 3.

**Figure 4:**
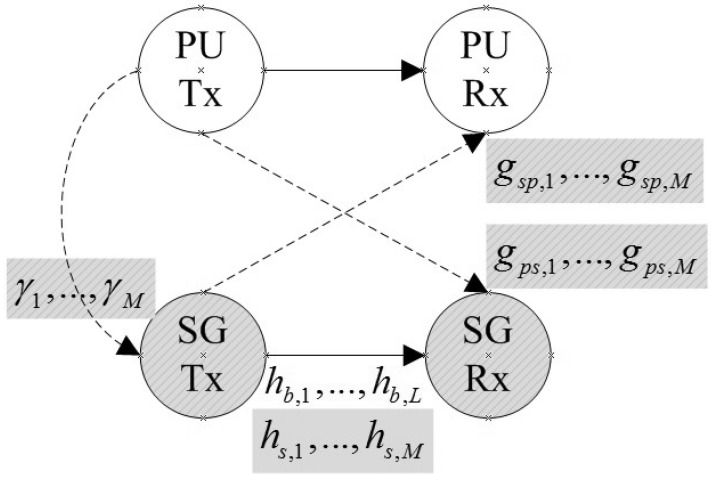
System model.

During the spectrum-sensing slot, the received SNRs of the PUs at the SG transmitter on the *M* cognitive channels are $\gamma_{1},\ldots ,\gamma_{M}$. The values of the received SNRs can be estimated by several methods and can be conducted by the HGWs and then broadcast to the NGWs via a control channel [18,19]. During the transmission slot, between the SG transmitter and the SG receiver, the channel gains of *L* original channels and *M* cognitive channels are $h_{b,1},\ldots ,h_{b,L}$ and $h_{s,1},\ldots ,h_{s,M}$. The channel gains between the SG transmitter and the PU receiver and the channel gains between the SG receiver and the PU transmitter of the *j* cognitive channel are $g_{sp,j}$ and $g_{ps,j}$(j=1,…,M). All channel gains were assumed to be block fading. The noise variances at the SG receiver of *L* original channels and *M* cognitive channels are $\sigma_{b,1}^{2},\ldots \sigma_{b,L}^{2}$ and $\sigma_{s,1}^{2},\ldots ,\sigma_{s,M}^{2}$. The system model is shown in Figure 4, where Tx denotes the transmitter and Rx denotes the receiver, while the solid lines denote the real communication links and the dashed lines denote the interference links.

Assume the transmission power constraint of the SG transmitter is *P*. In interweave CR, the powers allocated to the *L* original channels and *M* cognitive channels are $P_{bt,1},\ldots ,P_{bt,L}$ and $P_{st,1},\ldots ,P_{st,M}$. In underlay CR, the transmitted powers of the PUs are $P_{pu,1},\ldots ,P_{pu,M}$, and the interference limits for the PUs’ receivers are $\Psi_{1},\ldots ,\Psi_{M}$. The powers allocated to the *L* original channels are still $P_{bt,1},\ldots ,P_{bt,L}$. However, for the *M* cognitive channels, a two-level power allocation scheme is adopted. That is, under the absences of the PUs, high-level powers $P_{st,1}^{h},\ldots ,P_{st,M}^{h}$ are allocated, while under the presences of the PUs, low-level powers $P_{st,1}^{l},\ldots ,P_{st,M}^{l}$ are allocated.

Assume $\pi_{0,j}$ and $\pi_{1,j}$ are the probabilities that the *j*-th cognitive channel is unused and used by the PUs and the minimum target detection probability $P_{d0,j}$ is required for the *j*-th channel. Then, our objective is to find the optimal sensing time and power allocation strategy to maximize the data rate while satisfying all the constraints for the CR-enabled SG communication network.

## 3. Proposed Method for the Interweave-Cognitive-Radio-Enabled Smart Grid Network

In the interweave-CR-enabled SG, the SG user can only access the cognitive channels when the PUs are absent. Thus, the effective transmissions over the *M* cognitive channels occur only if the SG user can properly detect the absences of the PUs when the PUs are really absent. Hence, the data rate of the *M* cognitive channels is:

(5) $$R_{s}^{{}^{\prime }}=\sum_{j=1}^{M}B_{s,j}\log_{2}\left(1+\frac{P_{st,j}h_{s,j}^{2}}{\sigma_{s,j}^{2}}\right)\pi_{0,j}\left(1-P_{fa0,j}\right)$$

where $P_{fa0,j}=\frac{1}{2}erfc\left(erfc^{-1}\left(2P_{d0,j}\right)\sqrt{1+2\gamma }+\frac{\gamma \sqrt{N}}{2}\right)$.

The data rate should be averaged over time. According to Figure 2, the original channels and cognitive channels share one antenna so that all channels can only perform transmission processing during the transmission slot. Hence, $R_{s}^{{}^{\prime }}$ becomes:

(6) $$R_{s}^{{}^{\prime }}=\frac{T-\tau }{T}\sum_{j=1}^{M}B_{s,j}\log_{2}\left(1+\frac{P_{st,j}h_{s,j}^{2}}{\sigma_{s,j}^{2}}\right)\pi_{0,j}\left(1-P_{fa0,j}\right)$$

The data rate of the *L* original channels can also be obtained:

(7) $$R_{b}=\frac{T-\tau }{T}\sum_{i=1}^{L}B_{b,i}\log_{2}\left[1+\frac{P_{bt,i}h_{b,i}^{2}}{\sigma_{b,i}^{2}}\right]$$

As a result, the total data rate *R* is:

(8) $$R=R_{b}+R_{s}^{{}^{\prime }}$$

Considering the transmission power constraint *P*, the problem can be formulated as:

(9) $$\begin{array}{c} \underset{\tau ,P_{\mathrm{bt}},P_{\mathrm{st}}}{\arg\;\min}\;\;\;\;\;\;-R\;\;\;s.t.\;\left\{\begin{array}{c} \sum_{i=1}^{L}P_{bt,i}+\sum_{j=1}^{M}P_{st,j}=P\\ 0\leq \tau \leq T \end{array}\right. \end{array}$$

where $P_{\mathrm{bt}}=\left[P_{bt,1},\ldots ,P_{bt,L}\right],P_{\mathrm{st}}=\left[P_{st,1},\ldots ,P_{st,M}\right]$. The objective function is −R in (9), because finding the maximum of *R* is finding the minimum of −R.

Taking the second derivatives of −R with respect to the variables $P_{bt,i}$ and $P_{st,j}$, we have:

(10) $$\frac{\partial^{2}\left(-R\right)}{\partial P_{bt,i}^{2}}=\frac{\left(T-\tau \right)B_{b,i}h_{b,i}^{4}}{T\ln2{\left(\sigma_{b,i}^{2}+P_{bt,i}h_{b,i}^{2}\right)}^{2}}\geq 0$$

(11) $$\frac{\partial^{2}\left(-R\right)}{\partial P_{st,j}^{2}}=\frac{\left(T-\tau \right)B_{s,j}\pi_{0,j}\left(1-P_{fa0,j}\right)h_{s,j}^{4}}{T\ln2{\left(\sigma_{s,j}^{2}+P_{st,j}h_{s,j}^{2}\right)}^{2}}\geq 0$$

As a result, −R is convex with respect to $P_{bt,i}$ and $P_{st,j}$, but it is not convex with respect to the sensing time τ. Thus, (9) cannot be solved directly by using convex optimization methods. However, taking into account that the optimal sensing time τ is within the range of 0–*T*, so that it can be obtained by exhaustive search, therefore, assuming τ is fixed, by using the Lagrange multiplier method, we can obtain:

(12) $$\begin{array}{c} L\left(P_{\mathrm{bt}},P_{\mathrm{st}},\mu \right)=\frac{-(T-\tau )}{T}\sum_{i=1}^{L}B_{b,i}\log_{2}\left(1+\frac{P_{bt,i}h_{b,i}^{2}}{\sigma_{b,i}^{2}}\right)-\frac{T-\tau }{T}\sum_{j=1}^{M}\log_{2}\left(1+\frac{P_{st,j}h_{s,j}^{2}}{\sigma_{s,j}^{2}}\right)\\ \;\;\;\;\;\;\;\;\;\times B_{s,j}\pi_{0,j}\left(1-P_{fa0,j}\right)+\mu \left(\sum_{i=1}^{L}P_{bt,i}+\sum_{j=1}^{M}P_{st,j}-P\right) \end{array}$$

Taking the first derivatives of $L(P_{\mathrm{bt}},P_{\mathrm{st}},\mu )$ with respect to $P_{bt,i}$ and $P_{st,j}$ and then setting them to zero, we have:

(13) $$\begin{array}{c} \frac{\partial L(P_{\mathrm{bt}},P_{\mathrm{st}},\mu )}{\partial P_{bt,i}}=\mu -\frac{\left(T-\tau \right)B_{b,i}h_{b,i}^{2}}{T\ln2\left(\sigma_{b,i}^{2}+P_{bt,i}h_{b,i}^{2}\right)}=0\\ \Rightarrow P_{bt,i}=\frac{\left(T-\tau \right)B_{b,i}}{\mu T\ln2}-\frac{\sigma_{b,i}^{2}}{h_{b,i}^{2}} \end{array}$$

(14) $$\begin{array}{c} \frac{\partial L(P_{\mathrm{bt}},P_{\mathrm{st}},\mu )}{\partial P_{st,j}}=\mu -\frac{\left(T-\tau \right)B_{s,j}\pi_{0,j}h_{s,j}^{2}\left(1-P_{fa0,j}\right)}{T\ln2\left(P_{st,j}h_{s,j}^{2}+\sigma_{s,j}^{2}\right)}=0\\ \Rightarrow P_{st,j}=\frac{\left(T-\tau \right)B_{s,j}\pi_{0,j}\left(1-P_{fa0,j}\right)}{\mu T\ln2}-\frac{\sigma_{s,j}^{2}}{h_{s,j}^{2}} \end{array}$$

Taking the first derivative of $L(P_{\mathrm{bt}},P_{\mathrm{st}},\mu )$ with respect to μ and setting it to zero yields:

(15) $$\sum_{i=1}^{L}P_{bt,i}+\sum_{j=1}^{M}P_{st,j}=P$$

Applying (13) and (14) in (15), we can obtain:

(16) $$\frac{T-\tau }{\mu T\ln2}=\frac{P+\sum_{i=1}^{L}\frac{\sigma_{b,i}^{2}}{h_{b,i}^{2}}+\sum_{j=1}^{M}\frac{\sigma_{s,j}^{2}}{h_{s,j}^{2}}}{\sum_{i=1}^{L}B_{b,i}+\sum_{j=1}^{M}B_{s,j}\pi_{0,j}\left(1-P_{fa0,j}\right)}$$

Finally, substituting (16) into (13) and (14), the optimal power allocation scheme for a fixed τ can be obtained:

(17) $${\tilde{P}}_{bt,i}={\left\lfloor \frac{B_{b,i}\left(P+\sum_{i=1}^{L}\frac{\sigma_{b,i}^{2}}{h_{b,i}^{2}}+\sum_{j=1}^{M}\frac{\sigma_{s,j}^{2}}{h_{s,j}^{2}}\right)}{\sum_{i=1}^{L}B_{b,i}+\sum_{j=1}^{M}B_{s,j}\pi_{0,j}\left(1-P_{fa0,j}\right)}-\frac{\sigma_{b,i}^{2}}{h_{b,i}^{2}}\right\rfloor }^{+}$$

(18) $$\begin{array}{c} {\tilde{P}}_{st,j}={\left\lfloor \frac{\left(P+\sum_{i=1}^{L}\frac{\sigma_{b,i}^{2}}{h_{b,i}^{2}}+\sum_{j=1}^{M}\frac{\sigma_{s,j}^{2}}{h_{s,j}^{2}}\right)B_{s,j}\pi_{0,j}\left(1-P_{fa0,j}\right)}{\sum_{i=1}^{L}B_{b,i}+\sum_{j=1}^{M}B_{s,j}\pi_{0,j}\left(1-P_{fa0,j}\right)}-\frac{\sigma_{s,j}^{2}}{h_{s,j}^{2}}\right\rfloor }^{+} \end{array}$$

where the symbol ${\left\lfloor x\right\rfloor }^{+}$ denotes max(0,x).

Finally, we propose the optimal multiband spectrum sensing and power allocation method for interweave-CR-enabled SG in the following table (Algorithm 1).
**Algorithm 1** Optimal multiband spectrum sensing and power allocation method for interweave-CR-enabled SG communication.1:**for** each τ in 0 to *T* **do**-According to the given target detection probability $P_{d0,j}$(j=1,...,M), compute the false alarm probability $P_{fa0,j}$ for each channel in terms of (4).-Compute ${\tilde{P}}_{bt,i}$(i=1,...,L) for each original channel according to (17).-Compute ${\tilde{P}}_{st,j}$(j=1,...,M) for each cognitive channel according to (18).-Compute the data rate *R* according to (8) for the given τ.2:**end for**3:Find the maximum data rate *R*, and the optimal sensing time and power allocation are the corresponding parameters of the maximum *R*, that is:$$\left(\tau^{*},P_{\mathrm{bt}}^{*},P_{\mathrm{st}}^{*}\right)=\underset{\tau ,P_{\mathrm{bt}},P_{\mathrm{st}}}{\arg\max}R\left(\tau ,P_{\mathrm{bt}},P_{\mathrm{st}}\right).$$

## 4. Proposed Method for the Underlay-Cognitive-Radio-Enabled Smart Grid Network

In the underlay-CR-enabled SG, cognitive channels owned by the PUs can be shared with the SUs under certain interference limits. When the PU of a cognitive channel is absent and the SG user can properly detect the absence of the PU, the SG user could access this channel and transmit data with a high-level power $P_{st,j}^{h}$. In this case, the effective data rate of the *M* cognitive channels is:

(19) $$R_{s1}=\sum_{j=1}^{M}B_{s,j}\log_{2}\left(1+\frac{P_{st,j}^{h}h_{s,j}^{2}}{\sigma_{s,j}^{2}}\right)\pi_{0,j}\left(1-P_{fa0,j}\right)$$

When the PU of a cognitive channel is absent and the SG user cannot properly detect the absence of the PU, the SG user could access and transmit data with a low-level power $P_{st,j}^{l}$. In this case, the effective data rate of the *M* cognitive channels is:

(20) $$R_{s2}=\sum_{j=1}^{M}B_{s,j}\log_{2}\left(1+\frac{P_{st,j}^{l}h_{s,j}^{2}}{\sigma_{s,j}^{2}}\right)\pi_{0,j}P_{fa0,j}$$

When the PU of a cognitive channel is present and the SG user can properly detect the presence of the PU, the SG user could access and transmit data with power $P_{st,j}^{l}$. In this case, the effective data rate of the *M* cognitive channels is:

(21) $$R_{s3}=\sum_{j=1}^{M}B_{s,j}\log_{2}\left(1+\frac{P_{st,j}^{l}h_{s,j}^{2}}{g_{ps,j}^{2}P_{pu,j}+\sigma_{s,j}^{2}}\right)\pi_{1,j}P_{d0,j}$$

When the PUs are present and the SG user failed to detect the presences of the PUs, then the SG user would transmit data with the high-level power $P_{st,j}^{h}$, and unfortunately, a collision would occur. The effective data rate is zero in this case.

Therefore, for the *M* cognitive channels, the total averaged effective data rate in time is:

(22) $$\begin{array}{c} R_{s}=\frac{T-\tau }{T}\left[\sum_{j=1}^{M}B_{s,j}\log_{2}\left(1+\frac{P_{st,j}^{h}h_{s,j}^{2}}{\sigma_{s,j}^{2}}\right)\pi_{0,j}\left(1-P_{fa0,j}\right)+\sum_{j=1}^{M}B_{s,j}\log_{2}\left(1+\frac{P_{st,j}^{l}h_{s,j}^{2}}{\sigma_{s,j}^{2}}\right)\right.\\ \left.\;\;\;\;\;\;\;\times \pi_{0,j}P_{fa0,j}+\sum_{j=1}^{M}B_{s,j}\log_{2}\left(1+\frac{P_{st,j}^{l}h_{s,j}^{2}}{g_{ps,j}^{2}P_{PU,j}+\sigma_{s,j}^{2}}\right)\pi_{1,j}P_{d0,j}\right] \end{array}$$

For the *L* original channels, since they can be used without spectrum sensing, the effective data rate $R_{b}$ is the same as (7). As a result, the total effective data rate *R* is:

(23) $$R=R_{b}+R_{s}$$

Since a two-level power allocation scheme is adopted, the transmission power constraint *P* should be statistically averaged:

(24) $$\begin{array}{c} P=\sum_{i=1}^{L}P_{bt,i}+\sum_{j=1}^{M}\left[P_{st,j}^{h}\pi_{0,j}\left(1-P_{fa0,j}\right)\right.+P_{st,j}^{h}\pi_{1,j}\left(1-P_{d0,j}\right)+\\ \;\;\;\;\;\;\;\;\;\;\;\left.P_{st,j}^{l}\pi_{0,j}P_{fa0,j}+P_{st,j}^{l}\pi_{1,j}P_{d0,j}\right] \end{array}$$

Besides, the interference limit for the *j*-th PU receiver is:

(25) $$\begin{array}{c} g_{sp,j}^{2}P_{st,j}^{h}\pi_{1,j}\left(1-P_{d0,j}\right)+g_{sp,j}^{2}P_{st,j}^{l}\pi_{1,j}P_{d0,j}\leq \Psi_{j}\;\;\left(j=1,...,M\right) \end{array}$$

Finally, the problem can be formulated as (26), where $P_{\mathrm{bt}}=\left[P_{bt,1},\ldots ,P_{bt,L}\right]$, $P_{\mathrm{st}}^{h}=\left[P_{st,1}^{h},\ldots ,P_{st,M}^{h}\right]$, and $P_{\mathrm{st}}^{l}=\left[P_{st,1}^{l},\ldots ,P_{st,M}^{l}\right]$.

(26) $$\begin{array}{c} \;\;\;\;\;\;\;\;\;\;\;\;\;\;\;\;\underset{\tau ,P_{\mathrm{bt}},P_{\mathrm{st}}^{h},P_{\mathrm{st}}^{l}}{\arg\;\min}\;\;-R\\ s.t.\;\left\{\begin{array}{c} \sum_{i=1}^{L}P_{bt,i}+\sum_{j=1}^{M}\left[P_{st,j}^{h}\pi_{0,j}\left(1-P_{fa0,j}\right)+P_{st,j}^{h}\pi_{1,j}\left(1-P_{d0,j}\right)+P_{st,j}^{l}\pi_{0,j}P_{fa0,j}+P_{st,j}^{l}\pi_{1,j}P_{d0,j}\right]=P\\ g_{sp,j}^{2}P_{st,j}^{h}\pi_{1,j}\left(1-P_{d0,j}\right)+g_{sp,j}^{2}P_{st,j}^{l}\pi_{1,j}P_{d0,j}\leq \Psi_{j}\;\left(j=1,...,M\right)\\ 0\leq \tau \leq T \end{array}\right. \end{array}$$

Taking the second derivatives of −R with respect to the variables $P_{bt,i}$, $P_{st,j}^{h}$ and $P_{st,j}^{l}$$\left(i=1,\ldots ,L,j=1,\ldots ,M\right)$ the same as in Section 3, we can also have:

(27) $$\frac{\partial^{2}\left(-R\right)}{\partial P_{bt,i}^{2}}=\frac{\left(T-\tau \right)B_{b,i}h_{b,i}^{4}}{T\ln2{\left(\sigma_{b,i}^{2}+P_{bt,i}h_{b,i}^{2}\right)}^{2}}\geq 0$$

(28) $$\frac{\partial^{2}\left(-R\right)}{\partial {\left(P_{st,j}^{h}\right)}^{2}}=\frac{\left(T-\tau \right)B_{s,j}\pi_{0,j}\left(1-P_{fa0,j}\right)h_{s,j}^{4}}{T\ln2{\left(\sigma_{s,j}^{2}+P_{st,j}^{h}h_{s,j}^{2}\right)}^{2}}\geq 0$$

(29) $$\begin{array}{c} \frac{\partial^{2}\left(-R\right)}{\partial {\left(P_{st,j}^{l}\right)}^{2}}=\frac{\left(T-\tau \right)B_{s,j}\pi_{0,j}P_{fa0,j}h_{s,j}^{4}}{T\ln2{\left(\sigma_{s,j}^{2}+P_{st,j}^{l}h_{s,j}^{2}\right)}^{2}}+\frac{\left(T-\tau \right)B_{s,j}\pi_{1,j}P_{d0,j}h_{s,j}^{4}}{T\ln2\left(g_{ps,j}^{2}P_{pu,j}+\sigma_{s,j}^{2}+P_{st,j}^{l}h_{s,j}^{2}\right)}\geq 0 \end{array}$$

Hence, −R is convex with respect to $P_{bt,i}$, $P_{st,j}^{h}$, and $P_{st,j}^{l}$, but it is still not convex with respect to τ. In order to solve this problem, we still use the exhaustive search method. Assuming that τ is fixed, by using the Lagrange multiplier method, we have:

(30) $$\begin{array}{c} L\left(P_{\mathrm{bt}},P_{\mathrm{st}}^{h},P_{\mathrm{st}}^{l},\mu ,K\right)=-R+\mu \left\{\sum_{i=1}^{L}P_{bt,i}+\right.\\ \;\sum_{j=1}^{M}\left[P_{st,j}^{h}\pi_{0,j}\left(1-P_{fa0,j}\right)+P_{st,j}^{h}\pi_{1,j}\left(1-P_{d0,j}\right)+\right.\\ \left.\;P_{st,j}^{l}\pi_{0,j}P_{fa0,j}+P_{st,j}^{l}\pi_{1,j}P_{d0,j}\right]-P\}+\sum_{j=1}^{M}\kappa_{j}\times \\ \;\left[g_{sp,j}^{2}P_{st,j}^{h}\pi_{1,j}\left(1-P_{d0,j}\right)+g_{sp,j}^{2}P_{st,j}^{l}\pi_{1,j}P_{d0,j}-\Psi_{j}\right] \end{array}$$

where μ and $K=[\kappa_{1},...,\kappa_{M}]$ are the Lagrangian multipliers.

According to the Lagrange duality theory (Chapter 5 of [20]), minimizing $L(P_{\mathrm{bt}},P_{\mathrm{st}}^{h},P_{\mathrm{st}}^{l},\mu ,K)$ is to maximize its Lagrange dual function:

(31) $$g\left(\mu ,K\right)=\min_{P_{\mathrm{bt}},P_{\mathrm{st}}^{h},P_{\mathrm{st}}^{l}}\;L\left(P_{\mathrm{bt}},P_{\mathrm{st}}^{h},P_{\mathrm{st}}^{l},\mu ,K|\mu ,K\right)$$

where $L(P_{\mathrm{bt}},P_{\mathrm{st}}^{h},P_{\mathrm{st}}^{l},\mu ,K|\mu ,K)$ denotes $L(P_{\mathrm{bt}},P_{\mathrm{st}}^{h},P_{\mathrm{st}}^{l},\;$μ,K) with fixed μ and K. Then, the problem becomes:

(32) $$\max_{\mu ,K}g\left(\mu ,K\right)=\max_{\mu ,K}\min_{P_{\mathrm{bt}},P_{\mathrm{st}}^{h},P_{\mathrm{st}}^{l}}\;L\left(\left.P_{\mathrm{bt}},P_{\mathrm{st}}^{h},P_{\mathrm{st}}^{l},\mu ,K\right|\mu ,K\right)$$

Hence, when μ and K are fixed, by first taking the first derivatives of $L(P_{\mathrm{bt}},P_{\mathrm{st}}^{h},P_{\mathrm{st}}^{l},\mu ,K|\mu ,K)$ with respect to $P_{bt,i}$, $P_{st,j}^{h}$ and $P_{st,j}^{l}$ and then setting them to zero, the optimal $P_{bt,i}$, $P_{st,j}^{h}$ and $P_{st,j}^{l}$ can be obtained as follows:

(33) $$\begin{array}{c} P_{bt,i}={\left\lfloor \frac{\left(T-\tau \right)}{\mu T\ln2}-\frac{\sigma_{b,i}^{2}}{h_{b,i}^{2}}\right\rfloor }^{+} \end{array}$$

(34) $$\begin{array}{c} P_{st,j}^{l}={\left\lfloor \frac{C_{0,j}+\sqrt{C_{0,j}^{2}-4C_{1,j}}}{2}\right\rfloor }^{+} \end{array}$$

(35) $$P_{st,j}^{h}={\left\lfloor \frac{\left(T-\tau \right)B_{s,j}\pi_{0,j}\left(1-P_{fa0,j}\right)}{T\ln2\left[\mu \left(\pi_{0,j}\left(1-P_{fa0,j}\right)+\pi_{1,j}\left(1-P_{d0,j}\right)\right)+\kappa_{j}g_{sp,j}^{2}\pi_{1,j}\left(1-P_{d0,j}\right)\right]}-\frac{\sigma_{s,j}^{2}}{h_{s,j}^{2}}\right\rfloor }^{+}$$

where $C_{0,j}$ and $C_{1,j}$ are:

(36) $$C_{0,j}=\frac{\left(T-\tau \right)\left(\pi_{0,j}P_{fa0,j}+\pi_{1,j}P_{d0,j}\right)B_{s,j}}{T\ln2\left[\mu \left(\pi_{0,j}P_{fa0,j}+\pi_{1,j}P_{d0,j}\right)+\kappa_{j}g_{sp,j}^{2}\pi_{1,j}P_{d0,j}\right]}-\frac{2\sigma_{s,j}^{2}+g_{ps,j}^{2}P_{pu,j}}{h_{s,j}^{2}}$$

(37) $$C_{1,j}=\frac{\sigma_{s,j}^{2}g_{ps,j}^{2}P_{pu,j}+\sigma_{s,j}^{4}}{h_{s,j}^{4}}-\frac{\left(T-\tau \right)\left(\pi_{0,j}P_{fa0,j}g_{ps,j}^{2}P_{pu,j}+\pi_{0,j}P_{fa0,j}\sigma_{s,j}^{2}+\pi_{1,j}P_{d0,j}\sigma_{s,j}^{2}\right)B_{s,j}}{T\ln2\left[\mu \left(\pi_{0,j}P_{fa0,j}+\pi_{1,j}P_{d0,j}\right)+\kappa_{j}g_{sp,j}^{2}\pi_{1,j}P_{d0,j}\right]h_{s,j}^{2}}$$

Then, in the next step, the optimal values of μ and K should be found to maximize g(μ,K). However, it is difficult to obtain the analytical solutions by common mathematical manipulations. Here, the gradient descent method is used to solve this problem. According to the principles of the gradient descent method, the Lagrange multipliers μ and K can be updated as:

(38) $$\mu_{new}=\mu_{old}+\alpha \frac{\partial g}{\partial \mu },\;\;K_{new}=K_{old}+\alpha \frac{\partial g}{\partial K}$$

where $\frac{\partial g}{\partial \mu }$ and $\frac{\partial g}{\partial K}=\left[\frac{\partial g}{\partial \kappa_{1}},...,\frac{\partial g}{\partial \kappa_{M}}\right]$ are the partial derivatives of g(μ,K) with respect to μ and K, which are shown as follows:

(39) $$\begin{array}{c} \frac{\partial g}{\partial \mu }=P-\sum_{i=1}^{L}P_{bt,i}-\sum_{j=1}^{M}\left[P_{st,j}^{h}\pi_{0,j}\left(1-P_{fa0,j}\right)+P_{st,j}^{h}\pi_{1,j}\left(1-P_{d0,j}\right)\right.\\ \;\;\;\;\;\;\;\;\;\;\;\left.+P_{st,j}^{l}\pi_{0,j}P_{fa0,j}+P_{st,j}^{l}\pi_{1,j}P_{d0,j}\right] \end{array}$$

(40) $$\begin{array}{c} \frac{\partial g}{\partial \kappa_{j}}=\Psi_{j}-g_{sp,j}^{2}P_{st,j}^{h}\pi_{1,j}\left(1-P_{d0,j}\right)-g_{sp,j}^{2}P_{st,j}^{l}\pi_{1,j}P_{d0,j}\;\left(j=1,...,M\right) \end{array}$$

and α is the step size and can be determined via a line search of the following approach (Chapter 9 of [20]):

(41) $$\alpha^{k}=\underset{\alpha }{\arg\min}g\left(\mu +\alpha \frac{\partial g}{\partial \mu },\kappa +\alpha \frac{\partial g}{\partial \kappa }\right)$$

In summary, the optimal multiband spectrum sensing and power allocation method for underlay-CR-enabled SG is proposed in the following table (Algorithm 2).
**Algorithm 2** Optimal multiband spectrum sensing and power allocation method for underlay-CR-enabled SG communication.1:**for** each τ in 0 to *T* **do**2:    Initialize μ and K.3:    According to the given target detection probability $P_{d0,j}$(j=1,...,M), compute the false alarm probability $P_{fa0,j}$ for each channel in terms of (4).4:    **repeat**-Compute $P_{bt,i}$(i=1,...,L) or each original channel according to (33).-Compute $P_{st,j}^{l}$, $P_{st,j}^{h}$(j=1,...,M) for each cognitive channel according to (34) and (35).-Update μ and K according to (38).5:    **until** μ and K converge. Obtain the optimal ${\tilde{P}}_{bt,i}$, ${\tilde{P}}_{st,j}^{h}$, and ${\tilde{P}}_{st,j}^{l}$, and then, calculate the data rate *R* according to (23) for the given τ.6:**end for**7:Find the maximum data rate *R*, and the optimal sensing time and power allocation are the corresponding parameters of the maximum *R*, that is:$$\left(\tau^{*}\;,\;P_{\mathrm{bt}}^{*},P_{\mathrm{st}}^{h*},P_{\mathrm{st}}^{l*}\right)=\;\underset{\tau \;,\;P_{\mathrm{bt}}\;,\;P_{\mathrm{st}}^{h}\;,\;P_{\mathrm{st}}^{l}}{\arg\max}R\left(\tau \;,\;P_{\mathrm{bt}},P_{\mathrm{st}}^{h},P_{\mathrm{st}}^{l}\right)$$

## 5. Simulation Results

In this section, we present some simulation results to prove the validity of the proposed methods. We assumed the CR-enabled SG communication network consists of one original channel named $B_{b,1}$ with bandwidth 100 kHz and two cognitive channels named $B_{s,1}$ and $B_{s,2}$, with equal bandwidths of 8 MHz. In many real systems, the total bandwidth is usually divided into several narrowband sub-channels, such as Global System for Mobile communication (GSM) systems with 25 MHz bandwidth and 125 sub-channels, Narrowband Internet of Things (NB-IoT) systems with 180 kHz bandwidth and 12 sub-channels, and IEEE 802.11g systems with 16.25 MHz and 52 sub-channels, so the bandwidth of a single sub-channel usually ranges from tens of kHz to hundreds of kHz, and it is reasonable to assume the bandwidth of the original channel bought from the telecommunication operator is 100 kHz here. In addition, 6–8 MHz is the typical bandwidth of Television White Spaces (TVWS) [21] for CR usage. The PUs’ signals in $B_{s,1}$ and $B_{s,2}$ are assumed to be Gaussian distributed, and the probabilities that the cognitive channels are used by PUs, which are denoted by $\pi_{1,1}$, $\pi_{1,2}$, are assumed to be equal. According to [22], the activities of the primary users over TVWS can be measured and modeled in advance. Therefore, $\pi_{1,1}$ and $\pi_{1,2}$ can also be measured and assumed to be known in advance. During the spectrum-sensing process, the received SNRs of the PUs at the SG user on the two cognitive channels $\gamma_{1}$ and $\gamma_{2}$ are assumed to be identical. The noise variances of $B_{b,1}$, $B_{s,1}$, and $B_{s,2}$, which are denoted by $\sigma_{b,1}^{2}$, $\sigma_{s,1}^{2}$, and $\sigma_{s,2}^{2}$, are 1 W. The minimum target detection probabilities for the two cognitive channels $P_{d0,1}$ and $P_{d0,2}$ are also assumed to be identical. All the channel gains were assumed to be Rayleigh distributed with variance one. In the underlay-CR-enabled SG, the PUs’ transmitted powers $P_{pu,1}$,$P_{pu,2}$ are 1 W and the interference limits $\Psi_{1},\Psi_{2}$ are 15 W.

**Figure 5:**
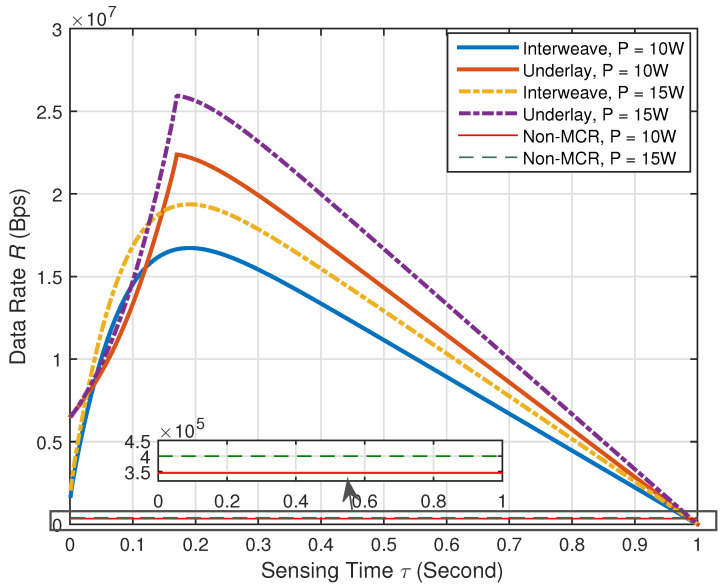
Sensing time versus data rate of the proposed methods in multiband-CR-enabled SG.

**Figure 6:**
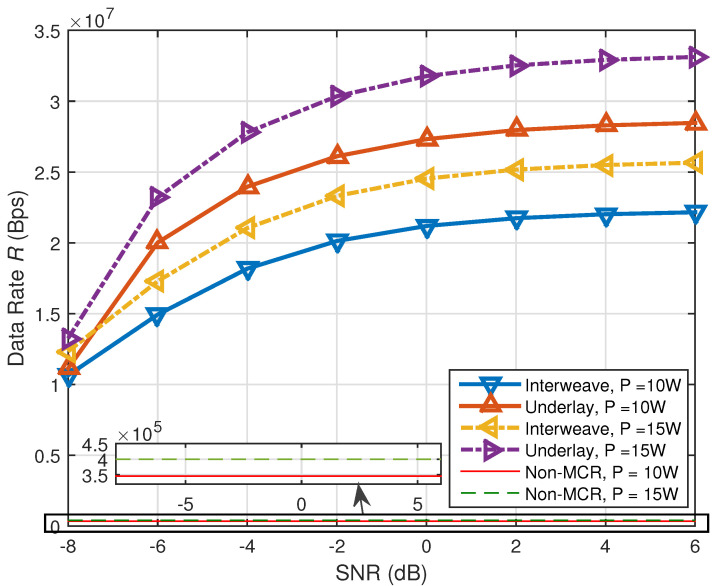
Received SNR versus data rate of the proposed methods in multiband-CR-enabled SG.

**Figure 7:**
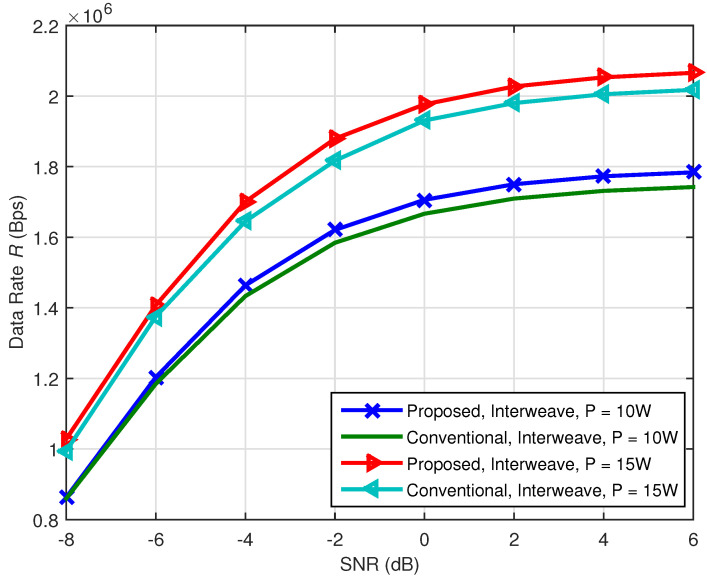
Comparison of the received SNR versus data rate curves between the proposed and conventional methods for interweave CR.

**Figure 8:**
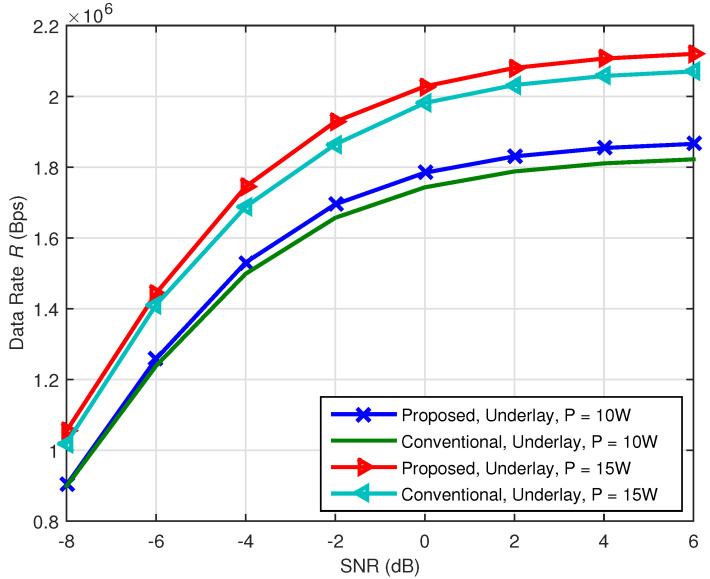
Comparison of the received SNR versus data rate curves between the proposed and conventional methods for underlay CR.

In Figure 5 and Figure 6, we make some comparisons between the proposed methods and the method without multiband CR technology and use “Non-MCR” to denote the data rate of the traditional method without multiband CR technology. In Figure 7 and Figure 8, we make some comparisons between the proposed methods and the method based on conventional CR network [14]. Note that, in Figure 5 and Figure 6, we assume $\pi_{1,1}=\pi_{1,2}=0.5$, $P_{d0,1}=P_{d0,2}=0.90$. Because no multiband CR technology was adopted, the SG user in such a communication network can only access the original channel $B_{b,1}$, and its data rate can be determined by $B_{b,1}$, $\sigma_{b,1}^{2}$ and the total transmission power constraint *P*. In Figure 7 and Figure 8, “conventional” denotes the method in [14] and: proposed: denotes our proposed methods. The communication network in [14] only has cognitive channels $B_{s,1}$ and $B_{s,2}$ and does not have the original channel $B_{b,1}$. Except for the original channel, all parameters of the conventional methods were set to be the same as the proposed methods.

Figure 5 shows the curves of sensing time τ versus data rate *R* of the proposed methods in multiband-CR-enabled SG. We set the time frame length T=1 s, the sampling interval $T_{s}=0.001$ s, the transmission power constraint P=10 W, 15 W, and the received SNRs of the PUs at the SG user on the two cognitive channels $\gamma_{1}=\gamma_{2}=-5$ dB. From Figure 5, for a given time frame length *T*, it can been seen that the data rate varies with the sensing time τ. Hence, it is necessary to find the optimal τ to maximize the data rate *R*. By using the exhaustive search method, the optimal sensing time τ is around 0.2 s for both interweave- and underlay-CR-enabled SG according to Figure 5. This is because the sensing time around 0.2 s is large enough to keep the target detection probability under the given conditions. Moreover, it can be seen that the data rate in underlay CR is usually higher than the data rate in interweave CR. This is because in underlay CR, the SG user can transmit data over the two cognitive channels under the interference limits even when the PUs are present, whereas it is strictly prohibited in interweave CR. Besides, for both interweave and underlay CR, when the total transmission power constraint *P* is increasing, the data rate is increasing. Figure 5 also shows that the multiband-CR-based method (either interweave CR or underlay CR) is far better than the method without multiband CR technology and thus proves the superiority of the proposed method. Therefore, it is very meaningful to adopt multiband CR technology in the SG communication network.

Figure 6 depicts the curves of the received PUs’ SNR versus data rate *R* of the proposed methods in multiband-CR-enabled SG. We set T=1 s, $T_{s}=0.001$ s, P=10 W, 15 W. As mentioned before, we assumed $\gamma_{1}=\gamma_{2}$. According to Figure 6, for both interweave and underlay CR, the data rates *R* are increasing while $\gamma_{1}$ and $\gamma_{2}$ are increasing, and they are all superior to the non-multiband-CR-based method. This is because $\gamma_{1}$ and $\gamma_{2}$ are larger; less sensing time τ is needed to meet the target detection probability, and thus, more time can be used for transmission. However, when $\gamma_{1}$ and $\gamma_{2}$ are large enough (larger than −2 dB), the increase of *R* becomes slow. This is because the necessary sensing time should be guaranteed to achieve the target detection probability and cannot be decreased any more. Again, we see that the data rate in underlay CR is higher than the data rate in interweave CR, having about a 2–3 dB advantage.

Figure 7 and Figure 8 give the comparison of the received SNR versus data rate curves for interweave and underlay CR between the conventional and proposed methods, respectively. We set the cognitive channel used probabilities to 0.85, that is $\pi_{1,1}=\pi_{1,2}=0.85$. It can be seen from Figure 7 and Figure 8 that the proposed methods are better than the conventional methods both for interweave and underlay CR. Besides, when the received SNR increases, the advantage becomes obvious. The main reason is similar to Figure 6. When the received SNR is higher, less sensing time is needed and more transmission time can be used.

As for the computational complexity, in interweave mode, the proposed method needs about 2L+3M+3 multiplications and 2L+3M additions to obtain ${\tilde{P}}_{bt,i}$ and about 2L+3M+5 multiplications and 2L+3M+1 additions to obtain ${\tilde{P}}_{st,j}$. Hence, the total computational complexity of the proposed method in interweave mode is about $(2L^{2}+3M^{2}+5ML+3L+5M)\tau$ multiplications and $2L^{2}+3M^{2}+5ML+M$ additions. The computational complexity of the conventional method in interweave mode is about (3M+5)Mτ multiplications and (3M+1)Mτ additions; in underlay mode, assuming the number of iterations in one round is $N_{T}\;\left(T=1,\ldots ,\tau \right)$, the proposed method needs about $\left(8M+1\right)N_{T}$ multiplications and $\left(L+6M+1\right)N_{T}$ additions to obtain μ and $7MN_{T}$ multiplications and $4MN_{T}$ additions to obtain K, then it needs about 4L multiplications and 2L additions to obtain $P_{bt,i}$, *M* square root operations, 2M multiplications, and 2M additions to obtain $P_{st,j}^{L}$, and 13M multiplications and 8M additions to obtain $P_{st,j}^{h}$. As a result, the total computational complexity of the proposed method in underlay mode is about Mτ square root operations, $\left(4L+47M\right)\tau +\sum_{N_{T}=1}^{\tau }\left(15M+1\right)N_{T}$ multiplications, and $\left(2L+23M\right)\tau +\sum_{N_{T}=1}^{\tau }\left(L+10M+1\right)N_{T}$ additions. The computational complexity of the conventional method in underlay mode is about Mτ square root operations, $46M\tau +\sum_{N_{T}=1}^{\tau }\left(15M+1\right)N_{T}$ multiplications, and $20M\tau +\sum_{N_{T}=1}^{\tau }\left(10M+1\right)N_{T}$ additions. Therefore, the computational complexity of the proposed method is higher than that of the conventional method. The computational complexities of the two methods are listed in Table 1.

## 6. Conclusions

As part of an IoT framework, smart grid applications require effective communication links among the HANs, NANs, and WANs. In this paper, a novel multiband-CR-based SG communication network architecture was proposed. Based on this, several joint spectrum sensing and power allocation methods were further proposed. By using convex optimization techniques, the optimal parameters such as the optimal sensing time and the optimal transmission power were found to maximize the data rate of multiband-CR-enabled SG while considering the target detection probabilities to the PUs. Simulations were presented to prove the correctness and the superiority of the proposed methods compared with the conventional methods.

## Figures and Tables

**Table 1:** Comparison of the computational complexities.

	Operations	Multiplications	Additions	Square Roots
Methods				
Proposed, Interweave		$(2L^{2}+3M^{2}+5ML+3L+5M)\tau$	$2L^{2}+3M^{2}+5ML+M$	0
Conventional, Interweave		(3M+5)Mτ	(3M+1)Mτ	0
Proposed, Underlay		$\left(4L+47M\right)\tau +\sum_{N_{T}=1}^{\tau }\left(15M+1\right)N_{T}$	$\left(2L+23M\right)\tau +\sum_{N_{T}=1}^{\tau }\left(L+10M+1\right)N_{T}$	Mτ
Conventional, Underlay		$46M\tau +\sum_{N_{T}=1}^{\tau }\left(15M+1\right)N_{T}$	$20M\tau +\sum_{N_{T}=1}^{\tau }\left(10M+1\right)N_{T}$	Mτ

## Data Availability

Not applicable.
